# Time to second cutaneous squamous cell carcinoma among Hispanic patients: A retrospective study

**DOI:** 10.1016/j.jdin.2025.11.031

**Published:** 2026-01-06

**Authors:** Kelly E. Owens, Marla A. Rodriguez Vazquez, Rebecca A. Zasloff, Beiyu Liu, Cynthia L. Green, Emily F. Cole, Melodi Javid Whitley

**Affiliations:** a Department of Dermatology, Duke University School of Medicine, Durham, North Carolina; b Duke University School of Medicine, Durham, North Carolina; c Department of Biostatistics & Bioinformatics, Duke University School of Medicine, Durham, North Carolina

**Keywords:** cutaneous squamous cell carcinoma, epidemiology, ethnicity, full body skin exam, Hispanic patients, Latina, Latino, nonmelanoma skin cancer, primary, risk factors, squamous cell carcinoma

*To the Editor:* Comprehensive studies on cutaneous squamous cell carcinoma (cSCC) in Hispanic populations are limited. Prior studies suggest differences in cSCC characteristics and prognosis for Hispanic/Latino patients compared to non-Hispanic groups.^1^, ^2^, ^3^, ^4^ Using a matched non-Hispanic cohort for comparison, this study assessed the incidence and timing of second cSCC development in Hispanic patients and associated risk factors.

We identified Hispanic patients with a first lifetime cSCC diagnosed between January 1, 2013 and December 31, 2020 using Duke’s electronic health records (Supplementary Table I, available via Mendeley at https://data.mendeley.com/datasets/3gg9f5rc9g/1). New primary tumors occurring ≥2 months after the first tumor were documented. Non-Hispanic patients were matched 2:1 by age at 1st diagnosis (±5 years), sex, smoking status, and year of 1st biopsy (±2 years). Kaplan–Meier methods compared second cSCC-free time between groups (log-rank test). Multivariable Cox proportional hazards models assessed if ethnicity was an independent risk factor, adjusting for immunosuppression, age at 1st cSCC biopsy, sex, smoking, tumor diameter, differentiation, and location. Additional models included interaction terms between ethnicity and each covariate (Supplementary Methods, available via Mendeley at https://data.mendeley.com/datasets/3gg9f5rc9g/1).

Among 65 Hispanic and 111 non-Hispanic patients (Supplementary Tables II and III, available via Mendeley at https://data.mendeley.com/datasets/3gg9f5rc9g/1), 24.6% of Hispanics and 55.0% of non-Hispanics developed a second cSCC (Supplementary Tables IV and V, available via Mendeley at https://data.mendeley.com/datasets/3gg9f5rc9g/1). Patients developing a second cSCC were predominantly male, white, nonsmokers, and in their mid-60s. First tumor characteristics were similar between groups. The median second cSCC-free time since the 1st SCC was estimated as 6.68 years for Hispanics versus 3.76 years for non-Hispanics (*P* = .036) (Fig 1). Stratification by immunosuppression status showed a significant difference in second cSCC-free time (*P* = .014) (Fig 2). The longer interval to second cSCC among Hispanics could reflect the combined effects of protective and nonprotective factors. Protective influences being genetically determined darker skin types, and nonprotective factors being provider biases in risk perception and follow-up along with barriers such as access, language, and cost.^1^, ^2^, ^3^, ^4^

**Fig 1:**
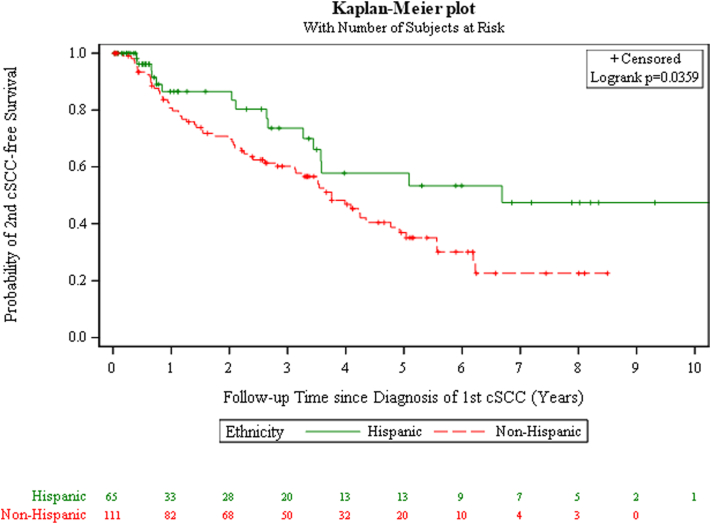
Squamous cell carcinoma. Kaplan–Meier plot of 2nd SCC-free stratified by ethnicity. For Hispanic patients, the 1-year, 3-year, and 5-year 2nd SCC-free survival rate was 86.5% (95% CI: 72.3% to 93.8%), 73.7% (55.9% to 85.2%), and 57.8% (38.2% to 73.3%), respectively. For non-Hispanic patients, the 1-year, 3-year, and 5-year 2nd SCC-free survival rate was 80.8% (71.8% to 87.1%), 60.2% (49.9% to 69.1%), and 36.9% (26.2% to 47.7%), respectively. Log-rank test shows that there is a significant difference in the 2nd SCC-free survival between Hispanic and non-Hispanic patients (*P* = .036). *CI*, Confidence interval; *SCC*, squamous cell carcinoma.

**Fig 2:**
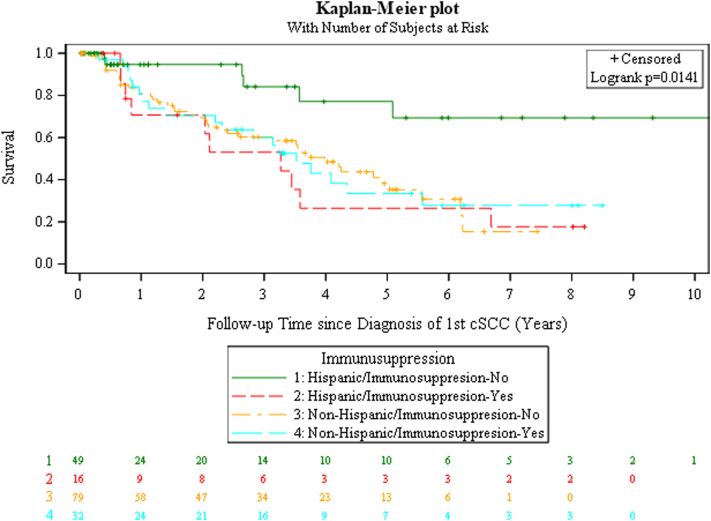
Squamous cell carcinoma. Kaplan–Meier plot of 2nd SCC-free survival stratified by immunosuppression and ethnic group. *SCC*, Squamous cell carcinoma.

After adjusting for other covariates, Hispanics had a 46% lower risk of developing a 2nd cSCC compared to non-Hispanics (hazard ratio: 0.54; 95% confidence interval: 0.30-0.98; *P* = .042). Only the interaction term between ethnicity and immunosuppression was significant (*P* = .039). Immunosuppressed Hispanics had a 4.62-fold increased hazard for second cSCC versus nonimmunosuppressed Hispanics (hazard ratio: 4.62; 95% confidence interval: 1.48-14.42; *P* = .008). No significant difference was observed in non-Hispanics by immunosuppression status (hazard ratio 1.21;95% confidence interval: 0.67-2.18; *P* = .53). Patients with darker skin types have a lower risk of cutaneous malignancy due to photoprotection from a higher amount of epidermal melanin, which filters more UV radiation.^5^ Perhaps the effects of immunosuppression become even more apparent when the competing risk of light skin types is lessened.

Limitations include retrospective nature, single institution, small sample size, and potential missing data from outside diagnoses. Matching may have resulted in a non-Hispanic cohort not fully representative of typical study populations.

Our findings suggest that Hispanic patients develop a second cSCC less frequently and later than non-Hispanics. Surveillance remains essential for both groups, with particular vigilance warranted for immunosuppressed Hispanic patients. Further studies are needed to guide follow-up strategies tailored to diverse populations.

## Conflicts of interest

None disclosed.
